# Dynamic Electromechanical Coupling of Piezoelectric Bending Actuators

**DOI:** 10.3390/mi7010012

**Published:** 2016-01-20

**Authors:** Mostafa R. A. Nabawy, William J. Crowther

**Affiliations:** School of Mechanical, Aerospace and Civil Engineering, The University of Manchester, Manchester M13 9PL, UK; bill.crowther@manchester.ac.uk

**Keywords:** piezoelectric, MEMS, actuators, electromechanical coupling, dynamics, resonance, actuation efficiency

## Abstract

Electromechanical coupling defines the ratio of electrical and mechanical energy exchanged during a flexure cycle of a piezoelectric actuator. This paper presents an analysis of the dynamic electromechanical coupling factor (dynamic EMCF) for cantilever based piezoelectric actuators and provides for the first time explicit expressions for calculation of dynamic EMCF based on arrangement of passive and active layers, layer geometry, and active and passive materials selection. Three main cantilever layer configurations are considered: unimorph, dual layer bimorph and triple layer bimorph. The actuator is modeled using standard constitutive dynamic equations that relate deflection and charge to force and voltage. A mode shape formulation is used for the cantilever dynamics that allows the generalized mass to be the actual mass at the first resonant frequency, removing the need for numerical integration in the design process. Results are presented in the form of physical insight from the model structure and also numerical evaluations of the model to provide trends in dynamic EMCF with actuator design parameters. For given material properties of the active and passive layers and given system overall damping ratio, the triple layer bimorph topology is the best in terms of theoretically achievable dynamic EMCF, followed by the dual layer bimorph. For a damping ratio of 0.035, the dynamic EMCF for an example dual layer bimorph configuration is 9% better than for a unimorph configuration. For configurations with a passive layer, the ratio of thicknesses for the passive and active layers is the primary geometric design variable. Choice of passive layer stiffness (Young’s modulus) relative to the stiffness of the material in the active layer is an important materials related design choice. For unimorph configurations, it is beneficial to use the highest stiffness possible passive material, whereas for triple layer bimorph configurations, the passive material should have a low stiffness. In all cases, increasing the transverse electromechanical coupling coefficient of the active material improves the dynamic EMCF.

## 1. Introduction

Piezoelectric bending actuators are an important class of micro electro-mechanical systems (MEMS) that find wide use in applications involving relatively large displacements in millimeter scale applications. Example dynamic applications include flapping wing propulsion [1,2,3,4,5,6,7], cooling fans [8,9,10], vibration control [11,12], and energy harvesters [13,14]. Dynamic operation at resonance with light damping significantly increases the achievable displacement of the actuator compared to the achievable static displacement for the same magnitude of electrical input. Whilst piezoelectric bending actuators are geometrically simple, the solution of the dynamic design problem is non-trivial, and, in recent years, there has been significant interest in providing theoretical, numerical and experimental contributions to dynamic characterization of these devices [13,14,15,16,17,18,19]. 

**Figure 1:**
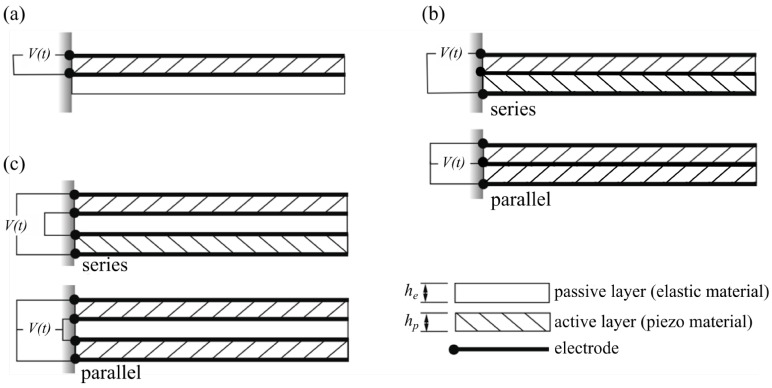
Piezoelectric bending actuators; illustration of the main configurations of practical interest. (**a**) Unimorph; (**b**) Double layer bimorph; (**c**) Triple layer bimorph. Sign of hatching direction illustrates sign of poling for piezo material.

A summary of the commonly used configurations of piezoelectric bending actuators is shown in Figure 1. It is assumed that actuators are made up of homogenous layers of active (piezoelectric) material or passive (elastic) material. The simplest viable configuration is comprised of one active layer and one passive layer and is referred to as a unimorph [20], Figure 1a. For configurations with two active layers, the general arrangement is one in which the two active layers are separated by an inner passive layer [21], Figure 1c. For the purposes of the present work, we will refer to this as a triple layer bimorph. In the limit when the thickness of the inner passive layer is reduced to zero, we reach the configuration shown in Figure 1b, which we will refer to as a double layer bimorph. Note that for both types of bimorphs shown, the active layers may be connected in series or parallel depending on the poling direction of the piezoelectric material.

One of the goals of actuator electromechanical design is to identify materials and configurations that maximize mechanical output for a given input electrical input, that is, the actuator is electromechanically efficient. Actuator electromechanical efficiency is usually measured using the electromechanical coupling factor (EMCF) denoted by *k*^2^, and the maximum energy transmission coefficient denoted by λ_max_ [20,22,23]. The EMCF is defined as the ratio of stored mechanical energy to the input electrical energy to the actuator, whilst the energy transmission coefficient is defined as the ratio of the output mechanical energy to the input electric energy [20,22,23]. The maximum energy transmission coefficient is a direct and single function of the EMCF [20]; thus, best configurations with respect to EMCF are also best with respect to the maximum energy transmission coefficient, and it is therefore sufficient to consider optimum actuator configurations based only on optimization of EMCF.

The static electromechanical coupling factor and the maximum energy transmission coefficient of unimorphs and double layer bimorphs have been assessed by Wang *et al.* [20] using static actuation constituent equations. It was shown that for double layer bimorphs, these measures are only a function of the piezoelectric material transverse coupling coefficient, *k*_31_, whereas for unimorphs, they are also a function of the Young’s modulus ratio and the thickness ratio of the actuator layers [20]. In a later contribution, the static actuation constitutive equations were derived for the triple layer bimorph configuration [21]; however its electromechanical coupling factor was not assessed. Maurini *et al.* [24] provided an extended Euler-Bernoulli beam model that considers the influence of 3D stresses and strains. The obtained coefficients of the static constituent equations for bimorph configurations were assessed against available standard models, and the achieved modeling improvement was demonstrated through comparisons with results from finite element simulations. The work [24] considered simply supported beams without analyzing other boundary conditions (e.g., cantilevers), and the electromechanical coupling factor was not addressed. The dynamic electromechanical coupling of unimorphs has been assessed by Chung *et al.* [4] based on the product of resonant frequency and vibration amplitude. The electromechanical coupling factor of unimorph actuators in dynamic operations has also been comprehensively assessed by the present authors [19]. It was found that the variation of dynamic EMCF with design variables is similar for both static and dynamic operation; however, for light damping, the dynamic EMCF will be an order of magnitude greater than for static operation.

The aim of the present work is to provide a comprehensive assessment of the electromechanical coupling characteristics of bimorph actuators in dynamic operation. Analytical expressions for double and triple layer bimorph actuators are derived in an explicit fashion allowing assessment of their dynamic actuation efficiency in a design context. The main contribution of this work is significantly improved understanding of the effect of configuration, material properties and operating conditions on the dynamic performance of bimorph actuators. The following section will provide a comprehensive theoretical model for the electromechanical coupling evaluation in dynamic operations. This will be followed by an analysis of the results from the theoretical model.

## 2. Dynamic Electromechanical Coupling Model

Following from references [20,22,23], a general expression for the electromechanical coupling factor (EMCF) can be written down as:

(1) $$k^{2}=\frac{D_{12}^{2}(l)}{D_{11}(l)D_{22}(l)}$$

where the *D* elements of the above expression are the symmetric matrix elements representing the set of constitutive equations of the actuator:

(2) $$\left[\begin{array}{c} \delta (l,t)\\ Q(l,t) \end{array}\right]=\left[\begin{array}{cc} D_{11}(l) & D_{12}(l)\\ D_{21}(l) & D_{22}(l) \end{array}\right]\left[\begin{array}{c} Fe^{i(\omega t)}\\ Ve^{i(\omega t)} \end{array}\right]$$

where δ, *F*, *Q* and *V* are the deflection, force, charge and voltage, respectively; *l* is the actuator tip position, *t* is time, and ω is the operation frequency. For details of the dynamic admittance matrix, see reference [19]. For the present work, the expression for the terms in the dynamic admittance matrix are re-written in a generic form that is independent of the configuration of the actuator as follows:

(3) $$D_{11}(l)=\sum_{n=1}^{\infty }X_{n}^{2}(l)\frac{1}{\rho AI_{n}}\frac{1}{\omega_{n}^{2}\sqrt{{\left(1-r_{n}^{2}\right)}^{2}+{\left(2\zeta_{n}r_{n}\right)}^{2}}}$$

(4) $$D_{12}(l)=D_{21}(l)=-\sum_{n=1}^{\infty }X_{n}(l){X^{\prime }}_{n}(l)\frac{\alpha_{p}}{\rho AI_{n}}\frac{1}{\omega_{n}^{2}\sqrt{{\left(1-r_{n}^{2}\right)}^{2}+{\left(2\zeta_{n}r_{n}\right)}^{2}}}$$

(5) $$D_{22}(l)=\sum_{n=1}^{\infty }{\left({X^{\prime }}_{n}(l)\right)}^{2}\frac{\alpha_{p}^{2}}{\rho AI_{n}}\frac{1}{\omega_{n}^{2}\sqrt{{\left(1-r_{n}^{2}\right)}^{2}+{\left(2\zeta_{n}r_{n}\right)}^{2}}}+\left(\frac{1}{k_{31}^{2}}-1\right)Y_{p}d_{31}^{2}\frac{lb}{h_{E}}$$

where *n* indicates the *n*th vibration mode. Note that Equations (3)–(5) are derived based on the assumption of a uniform composite Euler–Bernoulli beam with very thin, perfectly conductive electrodes covering the entire top and bottom surfaces of the piezo layer [13,19]. Application of Euler–Bernoulli beam theory to composite beams is typically assumed to be valid for beams with length to thickness (aspect) ratios above 30 [25]. This is typically the case for many practical actuator/harvester applications [13,14,16,17,18,19]. For geometrical configurations where 1D beam theory becomes inappropriate, a higher level model should be used, for example, see [26]. 

Table 1 provides the configuration parameters for Equations (3)–(5) that will be used throughout the current derivation. These include: the neutral axis position, $\bar{y}$, the rigidity, *YI*, the mass per unit length, ρ*A*, the voltage loading parameter, α*_p_*, and the active layers’ thickness defining the electric field, *h_E_* (*i.e.*, *E* = *V*/*h*_E_). In the above expressions, *d*_31_ is the piezoelectric constant (piezoelectric material property), *k*_31_ is the piezoelectric material transverse electromechanical coupling coefficient (piezoelectric material property), *b* is the actuator width, *h* is the thickness, *Y* is the Young's modulus, ρ is the material density and the subscripts *e* and *p* denote the elastic and piezoelectric layers respectively. Note that the unimorph expressions are the most complex due to its non-symmetric configuration. In addition, note that there is some analytical redundancy in that the expressions for the double layer bimorph case can be obtained either by substituting *h_e_* = *h_p_*, *Y_e_* = *Y_p_* and ρ*_e_* = ρ*_p_* in the unimorph expressions or by substituting *h_e_* = 0, *Y*_e_ = 0 and ρ*_e_* = 0 in the triple layer bimorph expressions.

**Table 1:** Configuration parameters for different actuators.

Parameter	Unimorph [13,19]	Double Layer Bimorph	Triple Layer Bimorph [14]
$\bar{y}$	$\frac{\left(\frac{Y_{e}}{Y_{p}}\right)h_{e}^{2}+2h_{e}h_{p}+h_{p}^{2}}{2\left(\left(\frac{Y_{e}}{Y_{p}}\right)h_{e}+h_{p}\right)}$	$h_{p}$	$h_{p}+\frac{h_{e}}{2}$
YI	$\frac{b}{3}\left[\begin{array}{l} Y_{e}\left(\begin{array}{l} 3h_{e}\bar{y}(\bar{y}-h_{e})+\\ h_{e}^{3} \end{array}\right)+\\ Y_{p}\left(\begin{array}{l} 3h_{p}(\bar{y}-h_{e})\times \\ (\bar{y}-(h_{e}+h_{p}))+\\ h_{p}^{3} \end{array}\right) \end{array}\right]$	$\frac{2bY_{p}h_{p}^{3}}{3}$	$\frac{2b}{3}\left[\begin{array}{l} Y_{e}\left(\frac{h_{e}^{3}}{8}\right)+\\ Y_{p}\left(\begin{array}{l} {\left(h_{p}+\frac{h_{e}}{2}\right)}^{3}-\\ \frac{h_{e}^{3}}{8} \end{array}\right) \end{array}\right]$
ρA	$b(\rho_{e}h_{e}+\rho_{p}h_{p})$	$b(2\rho_{p}h_{p})$	$b(\rho_{e}h_{e}+2\rho_{p}h_{p})$
$\alpha_{p}$	$\frac{d_{31}Y_{p}b}{2}\left(h_{p}+2h_{e}-2\bar{y}\right)$	$\frac{d_{31}Y_{p}b}{2}h_{p}$	$\frac{d_{31}Y_{p}b}{2}\left(h_{p}+h_{e}\right)$
$h_{E}$	$h_{p}$	$2h_{p}$	$2h_{p}$

The parameters that control the vibration response in Equations (3)–(5) are the damping ratio, ζ_n_, and the frequency ratio, *r_n_ =* ω*/*ω*_n_*, where ω*_n_* is the natural frequency given by [27]:

(6) $$\omega_{n}=\frac{{\left(\beta_{n}l\right)}^{2}}{l^{2}}\sqrt{\frac{YI}{\rho A}}$$

where β*_n_* denotes the wave number. Note that the natural frequency for light damping is approximately the damped resonant frequency. 

Finally, the term *I_n_* is given by:

(7) $$I_{n}=\int_{0}^{l}X_{n}^{2}(x)dx$$

where *X_n_* is the mode shape function for fixed-free boundary conditions. For the current work, we define *X_n_* using [27]:

(8) $$X_{n}(x)=\left(\cosh(\beta_{n}x)-\cos(\beta_{n}x)\right)-\upsilon_{n}\left(\sinh(\beta_{n}x)-\sin(\beta_{n}x)\right)$$

where

(9) $$\upsilon_{n}=\frac{\sinh(\beta_{n}l)-\sin(\beta_{n}l)}{\cosh(\beta_{n}l)+\cos(\beta_{n}l)}$$

We choose this definition of the mode shape function over other variants in the literature because of the characteristic:

(10) $$I_{1}=l$$

and thus the so-called “generalized mass” [28] becomes the actual actuator mass at the first resonant frequency:

(11) $$\rho AI_{1}=\rho Al={\mathrm{mass}}_{\mathrm{act}}$$

This choice is of significance since it removes the need for numerical integration within the actuator design process and hence simple explicit analytical expressions can be obtained. Note also that the mode shape expression given by Equation (8) has the following useful characteristics:

(12) $$X_{1}(l)=2\mathrm{and}\;{X^{\prime }}_{1}(l)=2\beta_{1}\upsilon_{1}$$

where

(13) $$\beta_{1}=\frac{1.8751}{l}\mathrm{and}\;\upsilon_{1}=0.7341$$

We now return back to the dynamic matrix elements provided in Equations (3)–(5). For most piezoelectric MEMS applications, the fundamental vibration mode *r_n_* = *r*_1_ is of most interest as it delivers the maximum displacement gain for a given level of damping. We thus drop the summations in Equations (3)–(5). Making use of the mode shape properties given in Equations (12) and (13), Equations (3)–(5) reduce to:

(14) $$D_{11}(l)=4\frac{1}{{\mathrm{mass}}_{\mathrm{act}}}\frac{1}{\omega_{1}^{2}\sqrt{{\left(1-r_{1}^{2}\right)}^{2}+{\left(2\zeta_{1}r_{1}\right)}^{2}}}$$

(15) $$D_{12}(l)=D_{21}(l)=-\left(2\times \frac{2.753}{l}\right)\frac{\alpha_{p}}{{\mathrm{mass}}_{\mathrm{act}}}\frac{1}{\omega_{1}^{2}\sqrt{{\left(1-r_{1}^{2}\right)}^{2}+{\left(2\zeta_{1}r_{1}\right)}^{2}}}$$

(16) $$D_{22}(l)={\left(\frac{2.753}{l}\right)}^{2}\frac{\alpha_{p}^{2}}{{\mathrm{mass}}_{\mathrm{act}}}\frac{1}{\omega_{1}^{2}\sqrt{{\left(1-r_{1}^{2}\right)}^{2}+{\left(2\zeta_{1}r_{1}\right)}^{2}}}+\left(\frac{1}{k_{31}^{2}}-1\right)Y_{p}d_{31}^{2}\frac{lb}{h_{E}}$$

Using the above expressions, a general expression for the dynamic EMCF at the first resonant frequency (*i.e.*, *r*_1_ = 1) can be obtained as:

(17) $$k^{2}=\frac{D_{12}^{2}(l)}{D_{11}(l)D_{22}(l)}=\frac{B}{B+C}$$

where

(18) $$B={\left(\frac{2.753}{l}\right)}^{2}\frac{\alpha_{p}^{2}}{{\mathrm{mass}}_{\mathrm{act}}}\frac{1}{\omega_{1}^{2}\left(2\zeta_{1}\right)}$$

(19) $$C=\left(\frac{1}{k_{31}^{2}}-1\right)Y_{p}d_{31}^{2}\frac{lb}{h_{E}}$$

Note that the configuration expressions for the double layer and triple layer bimorphs presented in Table 1 are for the piezoelectric elements in series. For a parallel configuration, *D*_11_(*l*) will remain the same; however, ${D_{12}(l)|}_{\mathrm{parallel}}=2{D_{12}(l)|}_{\mathrm{series}}$, and ${D_{22}(l)|}_{\mathrm{parallel}}=4{D_{22}(l)|}_{\mathrm{series}}$ [14,21]. Therefore, using the EMCF expression Equation (1), it can be seen that the parallel EMCF remains the same as the series as required by fundamental physical considerations.

Now, in order to provide explicit expressions for the dynamic EMCF for the different bimorph configurations, the configuration expressions in Table 1 are substituted in Equations (17)–(19). Further to some mathematical manipulation, the following expression is obtained for the dynamic EMCF of the double layer bimorph:

(20) $$k^{2}=\frac{0.46}{0.46+\left(\frac{1}{k_{31}^{2}}-1\right)\left(2\zeta_{1}\right)}$$

Thus, the dynamic EMCF of a double layer bimorph is function of the PZT material transverse electromechanical coupling coefficient, *k*_31_, and the operation damping ratio, ζ_1_, only. Note that $k^{2}\to 1$ if either $k_{31}\to 1$ or $\zeta_{1}\to 0$ as required by fundamental physical considerations. In addition, it is directly inferred that there is no specific optimum values for *k*_31_ and ζ_1_ that would allow a maximum *k*^2^ value; that is, the higher the *k*_31_ value, the higher the dynamic EMCF, and the lower the ζ_1_ value, the higher the dynamic EMCF.

An explicit analytical expression for the dynamic EMCF is also obtained for the triple layer bimorph making use of its configuration properties in Table 1 in conjunction with Equations (17)–(19). With some mathematical effort, it can be shown that the dynamic EMCF for this configuration is given by:

(21) $$k^{2}=\frac{0.46\left(4R^{2}+4R+1\right)}{0.46\left(4R^{2}+4R+1\right)+\left(\frac{1}{k_{31}^{2}}-1\right)\left(2\zeta_{1}\right)\left(NR^{3}+3R^{2}+3R+1\right)}$$

where

(22) $$R=\frac{h_{e}}{2h_{p}}\;\mathrm{and}\;N=\frac{Y_{e}}{Y_{p}}$$

Note that for verification, Equation (21) returns to the double layer bimorph expression (Equation (20)) for the case *R* = 0, as required. Again, the higher the *k*_31_ value the higher the dynamic EMCF, and the lower the ζ_1_ value the higher the dynamic EMCF; however, the *k*^2^ value now depends on the layers’ thickness ratio, *R*, and the Young’s modulus ratio of the layers, *N*. Inspection of Equation (21) shows that the lower *N* values lead to higher *k*^2^ values; that is, an elastic material with lower Young’s modulus is favorable from a coupling point of view.

An expression for the optimum thickness ratio, *R*_opt_, can be derived using Equation (21). By differentiation with respect to *R* and equating the resultant expression to zero, the following condition is obtained:

(23) $$4NR^{4}+8NR^{3}+3NR^{2}-2R-1=0$$

There are four roots for the above equation; however, the only valid solution for the above equation that would allow a meaningful explicit expression for *R*_opt_ is:

(24) $$R_{\mathrm{opt}}=\frac{1}{4A}+A-\frac{1}{2}$$

where

(25) $$A={\left(\sqrt{{\left(\frac{1}{4N}-\frac{1}{8}\right)}^{2}-\frac{1}{64}}+\frac{1}{4N}-\frac{1}{8}\right)}^{\frac{1}{3}}$$

Thus, the optimal thickness ratio of elastic to active layer thickness exists for triple layer bimorphs, and this optimum value depends only on the ratio of material stiffness for the layers.

## 3. Results and Discussion

In this section, a demonstration of the insights from the derived model for the dynamic electromechanical coupling factor is presented for double and triple layer bimorphs. Note that, in all cases, the coupling factor is evaluated at the first resonant frequency.

### 3.1. Young's Modulus Ratio and Thickness Ratio Effects

**Figure 2:**
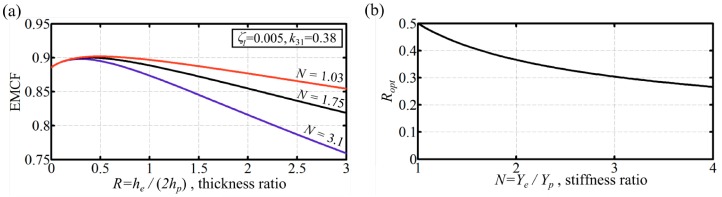
(**a**) triple layer bimorph dynamic EMCF as function of (passive/active) layer thickness ratio for different stiffness ratios. The damping ratio is 0.005. The active layer has a typical *k*_31_ = 0.38. For a piezoelectric material stiffness of 63 GPa, *N* = 3.1 represents steel (**blue**), *N* = 1.75 represents brass (**black**), and *N* = 1.03 represents aluminum (**red**); (**b**) dynamic operation optimum thickness ratio variation with the Young’s modulus ratio for triple layer bimorphs.

We consider here a triple layer bimorph with the material properties consistent with typical values for commercially available piezoelectric materials (*Y_p_* = 63 GPa and *k*_31_ = 0.38) [19]. Furthermore, we assume a damping ratio of 0.005 consistent with typical operational practice for piezoelectric MEMS applications [18,19]. Inspection of the results in Figure 2a confirms that, for triple layer bimorphs, there exists an optimum thickness ratio that maximizes the dynamic EMCF for given active and passive layer properties. Given that for zero thickness ratio (*R* = 0), the triple layer bimorph configuration becomes equivalent to the double layer bimorph, it can be seen that the triple layer configuration can achieve globally better dynamic EMCF values than the double layer configuration for a given set of material properties. Note also that the maximum achievable dynamic EMCF value for triple layer bimorphs increases with decreasing stiffness of the passive layer. For unimorphs, the behavior is the opposite: peak values of dynamic EMCF are increased by increasing the stiffness of the passive layer [19]. These behaviors are most evident when the damping ratio is high. The optimum layer thickness ratio, *R*_opt_, as a function of the stiffness ratio, *N*, is shown in Figure 2b. Remember that the value of *R*_opt_ is independent of both ζ_1_ and *k*_31_. For given piezoelectric material stiffness, increasing elastic layer stiffness (increasing *N*) means that the *R* value for peak dynamic EMCF reduces, *i.e.*, the thickness of the elastic layer must be reduced.

### 3.2. Damping Ratio and k_31_ Effects

**Figure 3:**
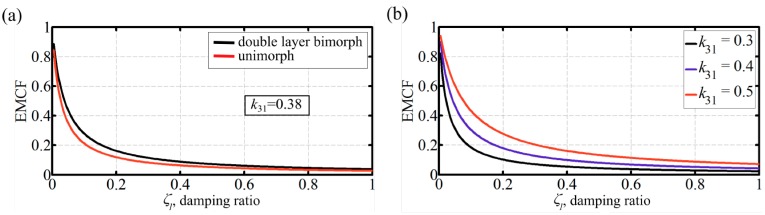
Double layer bimorph dynamic EMCF. (**a**) effect of damping ratio; *k*_31_ = 0.38. The EMCF of an optimum thickness unimorph with steel passive layer is shown for reference. Practically, this unimorph configuration can achieve the best EMCF values; thus, the superiority of double layer bimorphs against unimorphs is demonstrated; (**b**) effect of the PZT layer transverse electromechanical coupling coefficient for double layer bimorphs.

**Figure 4:**
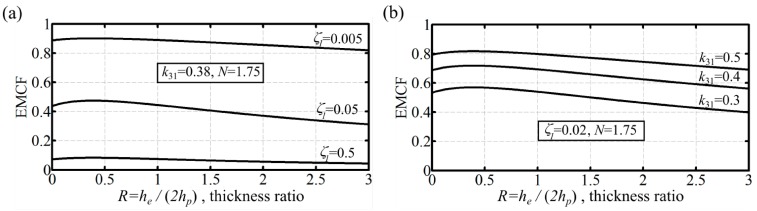
Triple layer bimorph dynamic EMCF as function of (passive/active) layer thickness ratio for a passive layer with a stiffness ratio of 1.75. (**a**) effect of damping ratio; *k*_31_ = 0.38; (**b**) effect of the PZT layer transverse electromechanical coupling coefficient; ζ_1_ = 0.02.

The effect of the damping ratio on dynamic EMCF for the double and triple layer bimorphs is shown in Figure 3a and Figure 4a, respectively. It can be confirmed for both cases that the dynamic EMCF values increase as the damping ratio decreases. In Figure 3a, the dynamic EMCF of an optimum thickness unimorph actuator with the same active material properties and an elastic layer made of steel is shown for reference. Given that unimorph peak values of dynamic EMCF are increased by increasing the stiffness of the passive layer [19], steel is used because it represents a practical higher end for the elastic material stiffness. Thus, this unimorph can be argued to demonstrate the highest practical dynamic performance. For the current demonstration at a damping ratio of 0.035, the dynamic EMCF for the double layer bimorph is 9% higher than that of the unimorph. This confirms that double layer bimorphs are capable of achieving better dynamic EMCF values compared to unimorphs. Varying the piezoelectric layer transverse electromechanical coupling coefficient, *k*_31_, has a similar effect as to that of damping ratio on the dynamic EMCF, Figure 3b and Figure 4b. A higher *k*_31_ (or a lower ζ_1_) will shift the dynamic EMCF curve up without affecting the value of *R*_opt_.

## 4. Conclusions

A novel analytical method for evaluation of the dynamic EMCF for cantilever bimorph piezoelectric actuators has been successfully developed in explicit form. This has been achieved through use of physical and mathematical insight to extend existing theoretical work. The model has been carefully parameterized to allow effective comparison of the main actuator multilayer topologies of engineering interest from unimorph to triple layer bimorph using a single set of equations. Several insights are obtained from the conducted analysis based on numerical evaluation of the model. For given material properties, it is shown that triple layer bimorphs can achieve higher dynamic EMCF values from simple double layer bimorphs. The benefit, however, depends on the damping ratio of the application. Having chosen an actuator topology, the subsequent step is selection of the actuator materials. For triple layer bimorphs, the passive material should have a low stiffness; however, for unimorphs, the highest stiffness possible passive material is recommended. For all actuator topologies, increasing the transverse electromechanical coupling coefficient of the active material improves the dynamic EMCF. As for the actuator geometry, configurations with a passive layer have an optimal thickness ratio which gives the best dynamic EMCF. This optimal thickness ratio is a function only of the Young’s modulus ratio of the elastic and active layers of the actuator. Increasing the elastic layer material Young’s modulus leads to an optimal thickness ratio corresponding to a thinner elastic layer. Finally, from an operation point of view, decreasing the damping ratio will directly increase the dynamic EMCF of the actuator.
